# Pharmacovigilance Practices by Healthcare Providers in Oncology: A Cross-Sectional Study

**DOI:** 10.3390/ph17060683

**Published:** 2024-05-26

**Authors:** Hadeel Alkofide, Haya M. Almalag, Mashael Alromaih, Lama Alotaibi, Njoud Altuwaijri, Noha Al Aloola, Jawza F. Alsabhan, Ghada A. Bawazeer, Lobna Al Juffali, Rihaf Alfaraj, Nora Alkhudair, Raniah Aljadeed, Rana Aljadeed, Lamya S. Alnaim

**Affiliations:** 1 Department of Clinical Pharmacy, College of Pharmacy, King Saud University, Riyadh 1145111, Saudi Arabia; halkofide@ksu.edu.sa (H.A.); halmalaq@ksu.edu.sa (H.M.A.); mashael.kalromaih@gmail.com (M.A.); nalaloola@ksu.edu.sa (N.A.A.); jawza@ksu.edu.sa (J.F.A.); gbawazeer@ksu.edu.sa (G.A.B.); laljaffali@ksu.edu.sa (L.A.J.); naalkhudair@ksu.edu.sa (N.A.); raaljadeed@ksu.edu.sa (R.A.); raljadeed@ksu.edu.sa (R.A.); 2 Drug Regulation Research Unit, College of Pharmacy, King Saud University, Riyadh 1145111, Saudi Arabia; 3 Department of Pharmaceutics, College of Pharmacy, King Saud University, Riyadh 1145111, Saudi Arabia; naltuwaijri@ksu.edu.sa (N.A.); ralfaraj@ksu.edu.sa (R.A.)

**Keywords:** pharmacovigilance, oncology, adverse drug reactions (ADRs), healthcare providers, patient safety, Saudi Arabia

## Abstract

Investigating pharmacovigilance (PV) practices among oncology healthcare providers (HCPs) is crucial for patient safety in oncology settings. This study aimed to assess the awareness, attitudes, and practices towards PV and identify barriers to effective adverse drug reaction (ADR) reporting for HCPs working in oncology-related settings. Employing a cross-sectional survey design, we collected data from 65 HCPs, focusing on their experiences with ADR reporting, education on ADR management, and familiarity with PV protocols. The results showed that about half of the responders were pharmacists. Around 58.9% of the respondents reported ADRs internally, and 76.9% had received some form of ADR-related education. However, only 38.5% were aware of formal ADR review procedures. Methotrexate and paclitaxel emerged as the drugs most frequently associated with ADRs. The complexity of cancer treatments was among the common reasons for the low reporting of ADRs by the study participants. The findings highlight the need for enhanced PV education and standardized reporting mechanisms to improve oncology care. We conclude that reinforcing PV training and streamlining ADR-reporting processes are critical to optimizing patient outcomes and safety in oncology, advocating for targeted educational interventions and the development of unified PV guidelines.

## 1. Introduction

Pharmacovigilance (PV) is crucial in healthcare and involves actions to identify and evaluate the effects of pharmacological treatments. This practice is essential to ensuring the safety and efficacy of drugs post marketing. The significance of PV was highlighted following the thalidomide disaster in the 1960s, a tragic event which underscored the potential risks and adverse effects associated with pharmaceutical products [1]. 

A major issue in handling drug reactions (ADRs) is the lack of awareness among healthcare providers (HCPs) regarding the procedures for reporting ADRs [2]. This limited understanding often leads to ADRs being under-reported, a problem highlighted in several research studies [3,4,5,6]. In the field of oncology, dealing with cancer medications adds complexity and urgency due to their potent nature [7]. The seriousness of ADRs in oncology is compounded by various factors. For example, HCPs might mistakenly attribute drug side effects to the cancer itself rather than its treatment, causing confusion about the drugs’ impacts [8]. Moreover, cancer treatment often involves tolerating levels of side effects due to the life-threatening nature of the disease and the aggressive treatment needed for management purposes [8]. As a result, prioritizing effectiveness tends to overshadow safety concerns, leading to a decreased reporting of ADRs in oncology settings [8]. This highlights the importance of enhancing education and systems to ensure the identification and reporting of ADRs, ultimately improving safety and efficacy in cancer treatments.

The advent of innovative treatments such as immunotherapies and targeted therapies has introduced fresh safety challenges, necessitating ongoing adjustments and enhancements to PV systems [9]. These novel therapeutic modalities, while significantly advancing the treatment landscape, also bring unique ADRs that require meticulous monitoring and reporting. The prevalent issue of under-reporting ADRs in the oncology sector represents a substantial risk to patient safety, as it can lead to the delayed recognition of critical safety concerns which are essential for informed treatment decisions [10,11]. The consequence of such delays is not trivial; it can adversely affect the therapeutic outcomes and overall well-being of patients with cancer [10,11]. To mitigate these risks and improve patient outcomes, it is crucial to advance PV practices within the oncology field [12]. This advancement involves not only the refinement of ADR-reporting systems but also the targeted education of HCPs [12]. By doing so, we can achieve a more comprehensive understanding of the safety profiles of oncology medications, ultimately ensuring that patients with cancer have access to safer and more effective treatment options.

In the study “A Survey of Adverse Event Reporting Practices Among US Healthcare Professionals”, key factors contributing to the under-reporting of ADRs were explored [13]. The complexity of attributing ADEs in polypharmacy contexts, the time constraints faced by HCPs, and the need for better integration and training in ADE-reporting systems were identified as key factors [13]. This research underscored the importance of systematic improvements in ADR reporting. 

To our knowledge, there has been no research focused on PV-reporting practices by HCPs managing cancer therapies in Saudi Arabia. Recent studies indicate a rise in the incidence of various cancer types in the region, with some cancers increasing threefold over the past decade [14,15]. This surge underscores the need to examine PV and ADR reporting among the growing oncology patient population. Our study aims to assess the awareness and knowledge of ADR reporting and PV practices among oncology HCPs. We intend to identify and rectify the deficiencies in PV practices to significantly enhance drug safety in oncology.

## 2. Results

### 2.1. Participant Characteristics

As of March 2022, we received 119 responses, and, out of those, 65 responders were eligible to enter this study. The excluded HCPs were 44.9% of our study participants, as they were working in settings other than oncology. Most participants were aged 25–35 years (56.9%), with a nearly equal distribution of sexes (49.2% female, 50.8% male). Most participants had 10–19 years of practice (36.9%) and worked in university hospitals (39.1%). Pharmacists represented the largest professional group (50.8%). The participants’ characteristics are presented in Table 1.

We compared the characteristics of the study subjects by sex and found no significant differences in most variables between male and female participants, except in the practice setting (*p* = 0.037). A higher percentage of female participants (31.2%) worked in Ministry of Health facilities compared to their male counterparts (12.1%). Conversely, a larger proportion of male subjects (21.2%) were employed in military hospitals than female subjects (12.5%). The data are presented in Table 1.

### 2.2. PV Knowledge and Reporting Practices

Most HCPs reported ADRs internally (58.9%), and a significant proportion (76.9%) received education related to ADRs. However, only 38.5% were familiar with a formal procedure for reviewing ADR reports (Table 2). When asked who most often submitted incidents reports at their organizations, 26% said that all HCPs equally submitted incident reports, while 22% indicated that this was the responsibility of nurses/physician’s assistants, and 15% stated that this was the responsibility of pharmacists. When comparing the results by sex, male and female participants had similar PV knowledge and reporting practices (Table 2).

**Figure 1:**
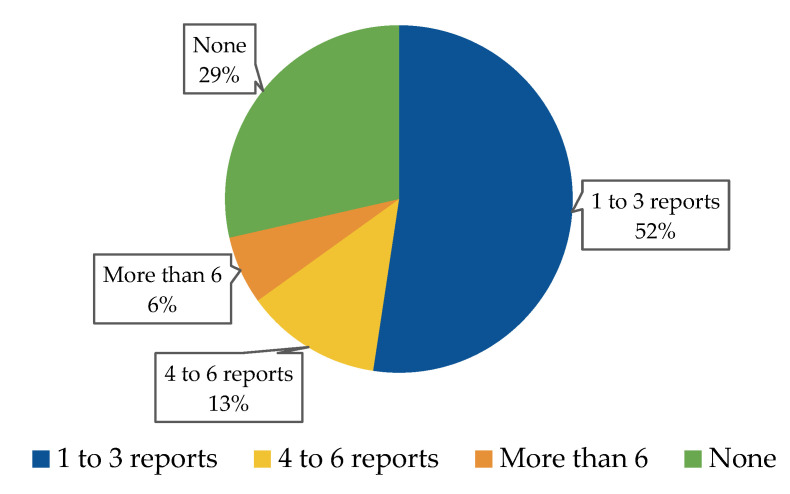
The average number of ADR incidents that HCPs reported on a monthly basis.

**Figure 2:**
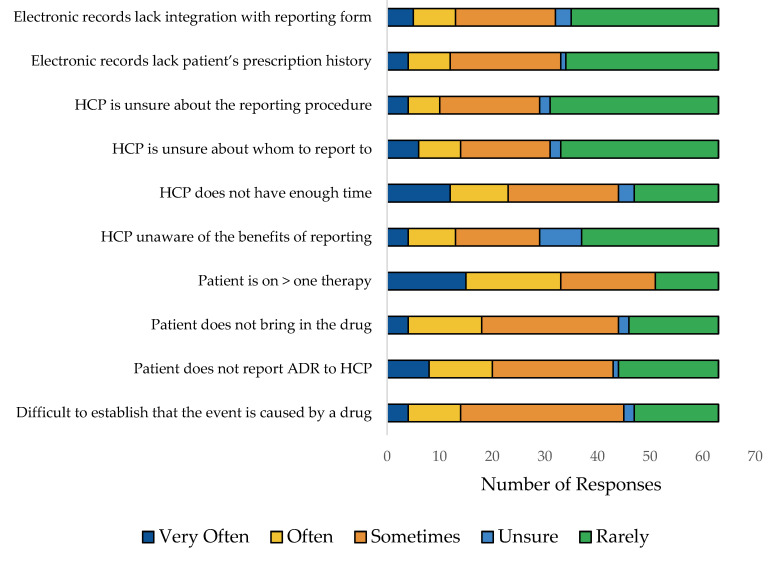
Reasons that may prevent HCPs from reporting ADRs.

On a monthly basis, more than 50% of respondents reported one to three incidents of ADRs (Figure 1). Among the various factors which may prevent HCPs from reporting ADRs, the most common explanation was that a patient was undergoing more than one treatment, making it difficult to determine which drug caused the ADR. The reasons that may prevent HCPs from reporting ADRs are presented in Figure 2. There were no significant differences between male and female subjects in the number of ADRs reported on a monthly basis (*p* = 0.404) 

### 2.3. PV-Reporting Process 

When the HCPs were asked where an ADR reporter from their organization might retrieve details about the primary suspect drug, most respondents (76.2%) stated that they used multiple methods to retrieve the information required. Of those, 50.1% used the incident report as one of the tools, while contacting a pharmacy was stated by 42.9% of the responders and patient charts, either electronic or paper-based, by 28.6%. 

About 62% of the responders used multiple methods to retrieve information related to the case details in an ADR. More than half of the respondents stated they would find the information they needed in the patient’s chart, followed by using the incident-reporting system. About 28% stated that they would contact the patient/caregiver to retrieve case-related information. 

The majority of study participants reported the full implementation of electronic systems at the centers where they worked (Table 3). The most implemented systems were the Computerized Physician Order Entry (COPE) system, followed by the Electronic Health Records (EHR)/Electronic Medical Records (EMR), the Electronic Medication Administration (EMA) (eMAR), and the electronic Incidents Reporting (IR) systems. The barcodes that enabled medication administration (BCMA) and the Barcode Medication Preparation Technologies (BCMP) systems were fully implemented in about 50% of the centers (Table 3). When comparing the results between male and female participants, no significant differences were observed in the PC-reporting processes between the sexes. 

When the participants were asked if there was a formal procedure for reviewing the reports submitted to the IR system (either paper-based or electronic) and deciding if they qualified for reporting to the SFDA PV program or the drug manufacturer, only 36.5% of the responders said yes. On the other hand, 6.3% said no, while almost 57% were unsure. 

### 2.4. ADRs Reported in Specific Oncology Medications

**Figure 3:**
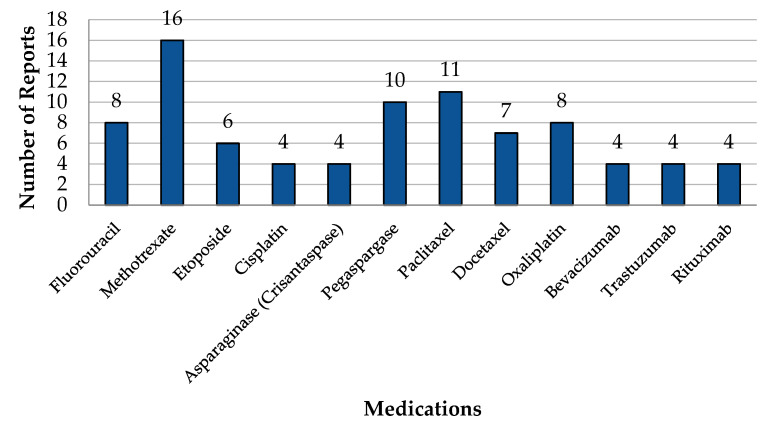
Medication for which healthcare providers have reported ADRs.

Methotrexate, followed by paclitaxel, was the most reported drug for ADRs by HCPs. Medications for which HCPs have reported ADRs are presented in Figure 3. The common ADRs encountered included nausea and vomiting (14.5%) and dermatological toxicity (11.4%). The most observed reaction type was unprovoked/unexpected (56.1%), and ADR severity was mostly categorized as Category 1 (30%). The characteristics of the ADRs reported for oncology drugs are presented in Table 4. 

### 2.5. HCP Perspective Regarding PV

**Figure 4:**
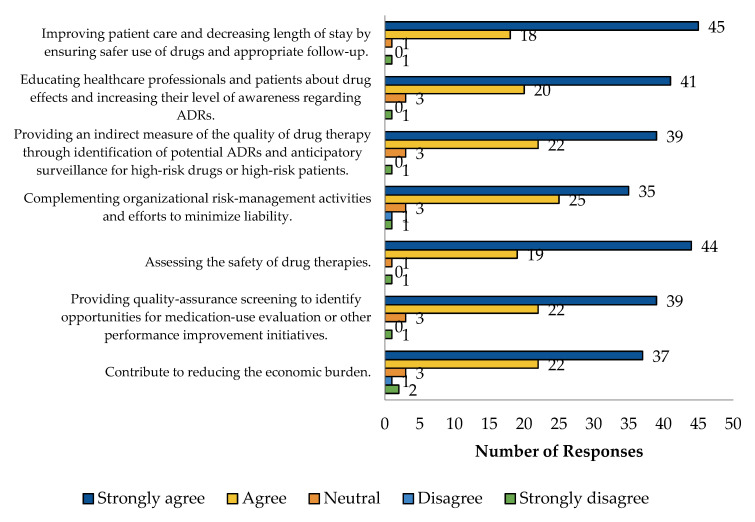
HCP perspectives regarding PV.

Most HCPs agreed that PV improved patient care and reduced hospital stays by ensuring safer drug use (45 respondents), educated HCPs and patients about drug effects and ADRs (41 respondents), and provided indirect measures of drug therapy quality (39 respondents). Additionally, PV supported organizational risk management (35 respondents), assessed drug safety (44 respondents), offered quality assurance in medication use (39 respondents), and contributed to reducing economic burdens (37 respondents). The overwhelming majority of responses either agreed or strongly agreed in all the categories. These data are presented in Figure 4. No differences were observed when comparing HCP perspectives regarding PV by sex.

## 3. Discussion

This study represents a pivotal exploration of PV practices among HCPs in oncology settings in Saudi Arabia, revealing both promising insights and critical areas for improvement. The commendable level of ADR-reporting education among HCPs, particularly pharmacists and those with over ten years of experience, underscores the foundational awareness necessary for effective PV. However, the persistently low reporting rates of ADRs highlight systemic issues within PV processes, mirroring global challenges in oncology PV [8,16,17].

The increased vulnerability of patients with cancer to ADRs can be attributed to the complexity of their condition and treatments [18,19]. This is further complicated by factors such as comorbidities, particularly in elderly patients, and the absence of complete safety and risk data for newly developed drugs or those used in pediatric populations [18]. The challenges in recognizing ADRs in oncology settings are often due to polypharmacy and under-reporting [18,20]. Nonetheless, it is crucial to acknowledge that ADR reporting in oncology settings has shown an upward trend over the years [10,21]. A recent study on PV for oncological prescriptions in patients aged 0–24 showed a significant increase in ADR reports, with off-label prescriptions being 3.4 times more likely to result in ADRs than on-label ones [21]. It was also found that 6.3% of prescriptions had errors and 18.2% near-misses, with 35.3% of 133 drug interactions causing ADRs [21]. This underscores the importance of active PV and a multidisciplinary team approach in enhancing safety, especially with off-label prescriptions.

Employing PV practices facilitates the early identification of potential ADRs [18,21]. A notable study delved into the ADRs, specifically leukopenia and thrombocytopenia, associated with CDK4/6 inhibitors in the treatment of breast cancer, by analyzing data from the European Spontaneous Adverse Event Reporting System [22]. Through an examination of 1611 individual case safety reports (ICSRs) from the EudraVigilance (EV) database, significant adverse events associated with palbociclib and ribociclib were uncovered [22]. The majority of these reports was classified as serious, particularly concerning leukopenia and thrombocytopenia [22]. This analysis highlights the crucial role of PV in not only identifying potential ADRs early on but also in distinguishing the toxicity profiles of CDK4/6 inhibitors within real-world settings, emphasizing the value of PV in enhancing patient safety and treatment outcomes [18,22].

In oncology, ADRs significantly impact patient outcomes [23,24,25]. Studies on voluntarily reported ADRs involving anticancer drugs highlight the prevalence among 41–60-year-olds, particularly female subjects, with skin issues and shivering as common symptoms associated with taxanes, targeted therapies, and platinum drugs [23]. Most of the ADRs were mild and preventable [23]. Another study reported that infections, nausea, and febrile neutropenia were frequent, involving drugs like platinum compounds and taxanes, with about 30.8% of reactions being preventable [24]. These findings emphasize the need for enhanced ADR surveillance and healthcare provider education to improve cancer care.

The phenomenon of ADR under-reporting in oncology settings extends beyond observational studies to encompass clinical trials in oncology [26,27]. Notably, research examining the disclosure of serious ADRs linked with targeted anticancer treatments in key phase III randomized clinical trials revealed a significant gap [26]. This study found that both the published accounts of these critical trials and the initial labeling of drugs frequently omit detailed information regarding serious ADRs associated with targeted oncology therapies [26].

The current study’s findings resonate with the broader literature, where under-reporting of ADRs is a prevalent issue, often attributed to a lack of feedback from management, legal concerns, and doubts regarding the value of reporting [28,29]. This aligns with the observed barriers in our study, including the complexity of cancer treatments and the ambiguity in distinguishing drug reactions from disease manifestations [13]. Importantly, our research underscores the necessity of targeted educational interventions to bridge the knowledge–application gap, particularly among less-experienced HCPs. Evidence from a Dutch study emphasizes the effectiveness of such interventions in improving reporting behaviors among specialist oncology nurses, suggesting a viable pathway to enhancing PV in oncology [30].

In exploring whether PV knowledge and practices vary by sex, we observed no significant differences between the male and female subjects in our study. Generally, gender differences in ADR reporting have not been extensively studied [31]. The literature suggests that women are more likely to report ADRs compared to men, which may be due to differences in how symptoms are perceived and attributed to medications [31,32]. Nonetheless, our findings did not show any differences in PV practices or ADR reporting between men and women. This absence of observed differences could potentially be attributed to the limited sample size of our participants. 

In our research, we demonstrated that, even with considerable awareness of PV, the actual reporting of ADRs continues to present difficulties, echoing findings from prior research [33,34]. For example, an investigation into the perceptions, knowledge, and behaviors of hospital pharmacists towards PV and ADR reporting in Najran, Saudi Arabia, utilized a questionnaire approach [33]. This investigation revealed that, although pharmacists generally grasp the concepts of PV and ADRs, there remains a noticeable deficiency in their knowledge [33]. The majority acknowledged the critical nature of ADR reporting as a professional obligation and the value of collaborative efforts in healthcare settings [33]. However, despite the acknowledged awareness of ADR-reporting mechanisms, obstacles such as the absence of forums for professional discussion and a shortfall in clinical knowledge act as significant impediments to reporting activities [33,35]. 

The literature does not uniformly present a positive outlook on the awareness and understanding of PV practices [36,37]. For instance, a comprehensive study conducted in Nepal, focusing on HCPs and their knowledge and perceptions of ADR reporting and PV, unveiled that a notable proportion of the respondents, with a majority being female, exhibited a lack of awareness about PV [36]. Among the various HCPs, pharmacists demonstrated somewhat-better knowledge [36]. The above study identified several significant barriers to ADR reporting, including the lack of active promotion by health authorities and uncertainties surrounding the proper procedures for reporting ADRs [36]. A recent systematic review has shown that attitudes regarding the reporting of ADRs continue to be the main determinants of under-reporting by HCPs [38].

This gap in understanding PV practices and the importance of ADR reporting is not confined to healthcare professionals but extends to the general public as well [37,39]. In a separate cross-sectional study conducted in Lithuania, the objective was to assess the general public’s knowledge, attitudes, and practices concerning ADR reporting [37]. The findings from this study indicated that, despite the Lithuanian public’s limited understanding of ADRs and PV, there existed a broadly positive attitude towards the significance of reporting ADRs [37]. Such findings have been confirmed by several other reports globally [39,40]. This discrepancy between the recognition of the importance of ADR reporting and the actual understanding and engagement in the PV process highlights a critical area for intervention.

These findings collectively emphasize the urgent need for meticulously designed educational campaigns and interventions aimed at boosting awareness and participation in ADR-reporting processes. Enhancing the collective understanding of ADRs, the mechanisms for reporting them, and the overarching concept of PV could significantly contribute to improving patient safety and the efficacy of healthcare delivery systems. By addressing these educational gaps through tailored programs and sustained awareness efforts, it is possible to build a more robust culture of PV that engages all stakeholders in the healthcare continuum.

The deployment of technological solutions within the realm of PV practices, as demonstrated by the widespread adoption of electronic reporting systems among the study participants, offers a pivotal avenue for enhancing the efficiency of PV processes. The utilization of EHRs alongside dedicated IR systems emerges as a key facilitator in achieving more streamlined, precise, and prompt ADR reporting [41,42]. Technological advancements enhance patient safety by swiftly identifying drug hazards. However, studies on drug–drug interaction (DDI) databases, which aid adverse drug reaction detection, show significant variability in identifying DDIs with new oral anticancer drugs, highlighting their limited reliability. This underscores the importance for healthcare providers to verify DDI data from multiple sources to ensure safe and effective drug therapies, especially in complex regimens involving new oral anticancer agents [43]. 

In addition, a recent and thorough review focusing on AI-driven PV has examined the capabilities of machine learning (ML) and deep learning (DL) techniques in detecting ADRs [44]. This analysis highlights the significant potential that AI holds in the field of PV, detailing both the strengths and challenges of the existing approaches and proposing directions for future research aimed at improving the extraction of ADRs from varied data sources [44]. The implications of this work for drug safety monitoring and healthcare outcomes are profound, signaling a move towards leveraging sophisticated computational techniques to advance the field of PV.

Our findings advocate for a multifaceted approach to fortify PV in oncology. Firstly, enhancing PV education, with a focus on practical applications, should be prioritized, leveraging the potential of continuous professional development and integrated learning modules. Secondly, simplifying and standardizing the reporting procedures can mitigate the perceived barriers, encouraging more proactive engagement from HCPs. Lastly, fostering a culture of open communication and feedback regarding ADR-reporting outcomes can demystify the process and reinforce its value within patient safety frameworks.

This study provides valuable insights but also presents several limitations that impact its interpretability and generalizability. Firstly, the use of self-reported data from HCPs may introduce bias, as participants could either overestimate their adherence to PV practices or under-report barriers to ADR reporting. Secondly, by focusing solely on oncology settings within a specific geographic area, the applicability of our findings to other regions and medical specialties is restricted. Thirdly, our use of convenient sampling methods, rather than random sampling, may further limit the generalizability of our results. Moreover, a significant limitation is our decision to go without a formal sample size calculation in favor of including all the potential participants, resulting in a relatively small sample size. This small sample may not capture the full variability in PV knowledge and practices across different settings or demographic groups, potentially limiting the generalizability of our conclusions and making the findings more susceptible to statistical errors. Finally, due to the cross-sectional survey design of our study and the nature of the data collected, specific details on ADRs were not collected.

Expanding the sample size in future research could enhance the reliability and applicability of the results. A larger and more diverse cohort would allow for a more detailed analysis of subgroups, such as differences based on years of experience, specific oncology subspecialties, or regional practices. Furthermore, increasing the sample size could provide a more powerful statistical basis for detecting significant differences and trends that our current study may not have been adequately posed to identify. Moreover, future cohort studies should specifically investigate ADRs in oncology settings, detailing the type of cancer, associated medications, dosages, and timing of administration to enhance the precision of the findings.

A significant strength of this study is its contribution to the scarce literature on PV practices in oncology, particularly within the context of Saudi Arabia. The detailed examination of factors influencing ADR reporting among HCPs provides valuable insights into both the challenges and opportunities for enhancing PV systems. Furthermore, this study’s methodology, involving a comprehensive survey and a robust analytical framework, ensures the reliability and relevance of the findings to stakeholders in PV and patient safety.

## 4. Materials and Methods

### 4.1. Study Design, Settings, and Population

We conducted a questionnaire-based, cross-sectional study from October 2021 to March 2022, targeting oncology HCPs—including physicians, pharmacists, technicians, and nurses—across hospitals in the Kingdom of Saudi Arabia.

### 4.2. Study Questionnaire

Our questionnaire, adapted with permission from Stergiopoulos et al. [13], was pilot-tested on a sample (*n* = 10) to assess the questions’ relevance and clarity. Revisions were made based on the feedback. The questionnaire encompassed four main themes: the reporting details, the reporting process, the responsible parties, and the system of reporting. It also gathered specific oncology-related responses concerning the most reported anticancer drug classes, ADR types, severity levels, and management actions taken.

### 4.3. Data Collection and Source

Data were systematically gathered using an online survey tool, specifically Google Forms, and disseminated across various channels, such as email, social media platforms, and professional networks, to ensure a wide reach. This included targeted distribution within specific networks, notably the Saudi Commission for Health Specialties and attendees of the Saudi Arabia Regional Oncology Pharmacy Conference. We used a convenient sampling technique in this study. Prior to participating in the survey, all the respondents were required to give their informed consent, ensuring that they were fully aware of this study’s nature and purpose and how their data would be used, aligning with ethical research practices. The survey was conducted anonymously, and no personal data were collected.

### 4.4. Study Variables

The demographic data collected included age, sex, professional role, years of experience, work setting, city, and employing institution. The survey also inquired about ADR-reporting processes, barriers to reporting, and knowledge of PV systems among HCPs. The questions also included those related to the common ADRs reported by HCPs, as well as the severity and category of these ADRs.

### 4.5. Data Presentation and Statistical Analysis

The data were presented using descriptive statistics, which included the computation of the frequencies and percentages for all the study variables, all of which were categorical. Excel version 16.57 was used to facilitate the organization and graphical representation of the data. Chi-Square tests measured differences between male and female participants across the study variables. A *p*-value of less than 0.05 was considered statistically significant. Analyses were conducted using the R statistical software version 4.3.1.

## 5. Conclusions

In conclusion, this study illuminates the critical facets of PV practices in oncology within Saudi Arabia, contributing valuable insights to the global discourse on improving drug safety monitoring. Addressing the identified gaps and leveraging technological advancements create a promising avenue to enhance PV efficacy, ultimately safeguarding patient outcomes in the complex landscape of cancer treatment.

## Figures and Tables

**Table 1:** Characteristics of the study participants (*n* = 65).

Characteristics	Overall Sample (*n* = 65)	Female (*n* = 32)	Male (*n* = 33)	*p*-Value *
Age in years:				0.772
25–35	37 (56.9%)	17 (53.1)	20 (60.6)	
36–45	17 (26.2%)	9 (28.1)	8 (24.2)	
46–55	8 (12.3%)	5 (15.6)	3 (9.1)	
56–65	3 (4.6%)	1 (3.1)	2 (6.1)	
66+	0 (0%)	0 (0%)	0 (0%)	
Years in practice:				0.934
Less than 1 year	4 (6.2%)	2 (6.2)	2 (6.1)	
1–4 years	16 (24.6%)	9 (28.1)	7 (21.2)	
5–9 years	15 (23.1%)	6 (18.8)	9 (27.3)	
10–19 years	24 (36.9%)	12 (37.5)	12 (36.4)	
20 years or more	6 (9.2%)	3 (9.4)	3 (9.1)	
Practice Setting:				0.037
Ministry of Health Facilities	14 (21.6%)	10 (31.2)	4 (12.1)	
Military Hospitals	11 (16.9%)	4 (12.5)	7 (21.2)	
Ministry of the Interior Hospitals	1 (1.6%)	0 (0.0)	1 (3.0)	
Referral Hospitals	6 (9.2%)	0 (0.0)	6 (18.2)	
University Hospitals	25 (38.4%)	15 (46.9)	10 (30.3)	
Private Facilities	8 (12.3%)	3 (9.4)	5 (15.2)	
Role:				0.070
Physician	7 (10.8%)	2 (6.2)	5 (15.2)	
Nurse	17 (26.2%)	13 (40.6)	4 (12.1)	
Pharmacist	33 (50.8%)	13 (40.6)	20 (60.6)	
Pharmacy technician	7 (10.8%)	3 (9.4)	4 (12.1)	
Other	1 (1.5%)	1 (3.1)	0 (0.0)	

Data presented as number (percentages). * Using the Chi-Square test.

**Table 2:** PV knowledge and reporting practices (*n* = 65).

Characteristics	Overall Sample (*n* = 65)	Female (*n* = 32)	Male (*n* = 33)	*p*-Value *
To which organization do HCPs report ADRs?				0.844
Internally (the healthcare organization where HCPs work)	38 (58.5)	18 (56.2)	20 (60.6)	
Ministry of Health	6 (9.2)	3 (9.4)	3 (9.1)	
Drug manufacturer	4 (6.1)	2 (6.2)	2 (6.0)	
SFDA PV Program	8 (12.3)	5 (15.6)	3 (9.1)	
Never reported	9 (13.8)	4 (12.5)	5 (15.2)	
Did HCPs receive ADR-related education?				0.602
Yes	50 (76.9)	26 (81.2)	24 (72.7)	
No	15 (23.1%)	6 (18.8)	9 (27.3)	
Are HCPs familiar with a formal procedure for reviewing reports submitted to the incident-reporting system?				0.561
Yes	25 (38.5%)	13 (40.6)	12 (36.4)	
No	4 (6.2%)	1 (3.1)	3 (9.1)	
Unsure	35 (53.8%)	18 (56.2)	17 (51.5)	
Not applicable	1 (1.5%)	0 (0.0)	1 (3.0)	
How do HCPs identify ADRs?				0.148
Temporal relationship between the onset of a drug therapy and the adverse reaction	21 (31.7%)	6 (18.8)	15 (45.5)	
There was a dechallenge or rechallenge	18 (27.0%)	12 (37.5)	6 (18.2)	
Signs and symptoms of ADRs	8 (12.7%)	4 (12.5)	4 (12.1)	
Laboratory tests	3 (4.8%)	1 (3.1)	2 (6.1)	
Patient complaint	15 (23.8%)	9 (28.1)	6 (18.2)	

Data presented as number (percentages). * Using the Chi-Square test.

**Table 3:** Extent of electronic systems implemented in the centers of the study participants (*n* = 65).

Electronic Systems’ Implementation	*n* (%)
Electronic Health Records (EHR)/Electronic Medical Records (EMR)	
Fully Implemented	51 (77.8%)
Not Implemented/Paper-based Methods Only	1 (1.6%)
Partially Implemented	12 (19.0%)
Unsure	1 (1.6%)
Barcode-enabled Medication Administration (BCMA)	
Fully Implemented	32 (49.2%)
Not Implemented/Paper-based Methods Only	6 (9.5%)
Partially Implemented	24 (36.5%)
Unsure	3 (4.8%)
Computerized Physician Order Entry (CPOE) System	
Fully Implemented	52 (79.4%)
Not Implemented/Paper-based Methods Only	1 (1.6%)
Partially Implemented	10 (15.9%)
Unsure	2 (3.2%)
BarCode Medication Preparation Technologies (BCMP)	
Fully Implemented	32 (49.2%)
Not Implemented/Paper-based Methods Only	6 (9.5%)
Partially Implemented	22 (33.3%)
Unsure	5 (7.9%)
Electronic Incident-Reporting (IR) System	
Fully Implemented	45 (69.8%)
Not Implemented/Paper-based Methods Only	1 (1.6%)
Partially Implemented	14 (22.2%)
Unsure	4 (6.3%)
Electronic Medication Administration Record (eMAR)	
Fully Implemented	45 (71.4%)
Partially Implemented	17 (25.4%)
Unsure	2 (3.2%)

**Table 4:** Types and characteristics of ADRs reported by the study participants (*n* = 65).

Reporting Processes	*n* (%)
Most encountered type of ADRs	
Nausea and vomiting	9 (14.5%)
Dermatological toxicities	8 (11.4%)
Febrile neutropenia	7 (10.4%)
Cardiovascular toxicity	6 (9.3%)
Diarrhea or constipation	5 (7.8%)
Fatigue	4 (6.2%)
Mucositis	4 (6.2%)
Thrombosis	3 (5.2%)
Infusion reactions	3 (5.2%)
Neuropathic pain	3 (5.2%)
Central venous catheters-related complications	2 (3.6%)
Infections	2 (3.6%)
Most observed reaction type *	
Unprovoked/Unexpected reaction	36 (56.1%)
Exaggerated pharmacological action	29 (43.9%)
Most observed level of ADR severity **	
Category 1	20 (30%)
Category 2	15 (23.3%)
Category 3	14 (21.1%)
Category 4	12 (18.9%)
Category 5	3 (4.4%)
Category 6	1 (2.2%)
Action taken most often once an ADR is detected	
Discontinue suspect medication(s)	17 (26.9%)
Treatment with medications	13 (20.7%)
Adjust dose, route, frequency	13 (20.7%)
Therapy held	11 (16.6%)
Switch to alternative agent	8 (11.7%)

* Definitions of exaggerated pharmacological activity and unexpected/unprovoked reactions: Exaggerated pharmacological activity occurs due to excessive modification of the activity of the primary pharmacological target beyond the point necessary for efficacy. An unexpected/unprovoked reaction occurs without an apparent cause or justification. ** Level of ADR severity defined using the following criteria: Category 1: Circumstances or processes that have the potential to cause ADR. Category 2: An event occurred, but the patient was not harmed. Category 3: An event occurred that resulted in the need for increased patient assessments but no change in vital signs and no patient harm. Category 4: An event occurred that resulted in the need for treatment and/or intervention and caused temporary patient harm. Category 5: An event occurred that resulted in initial or prolonged hospitalization, affected patient participation in an investigational drug study, and/or caused temporary patient harm. Category 6: An event occurred that resulted in permanent patient harm or a near-death event, such as anaphylaxis.

## Data Availability

The data that support the findings of this study are available from the corresponding author [LA] upon reasonable request.
